# Structure and Mechanism of *Pseudomonas aeruginosa* PA0254/HudA, a prFMN-Dependent Pyrrole-2-carboxylic Acid Decarboxylase Linked to Virulence

**DOI:** 10.1021/acscatal.0c05042

**Published:** 2021-02-17

**Authors:** Karl A. P. Payne, Stephen A. Marshall, Karl Fisher, Stephen E. J. Rigby, Matthew J. Cliff, Reynard Spiess, Diego M. Cannas, Igor Larrosa, Sam Hay, David Leys

**Affiliations:** † Manchester Institute of Biotechnology, University of Manchester, 131 Princess Street, Manchester M1 7DN, United Kingdom; § Department of Chemistry, University of Manchester, Chemistry Building, Oxford Road, Manchester M13 9PL, United Kingdom

**Keywords:** decarboxylase, enzyme mechanism, flavin chemistry, prFMN, *Pseudomonas aeruginosa*, quorum sensing, pyrrole-2-carboxylic acid

## Abstract

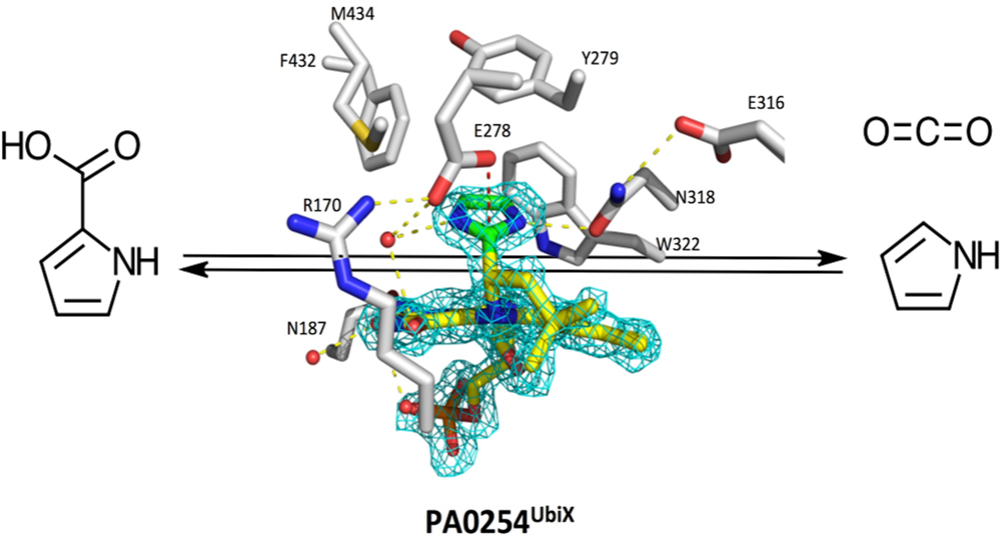

The UbiD family of reversible (de)carboxylases depends on the recently discovered prenylated-FMN (prFMN) cofactor for activity. The model enzyme ferulic acid decarboxylase (Fdc1) decarboxylates unsaturated aliphatic acids via a reversible 1,3-cycloaddition process. Protein engineering has extended the Fdc1 substrate range to include (hetero)aromatic acids, although catalytic rates remain poor. This raises the question how efficient decarboxylation of (hetero)aromatic acids is achieved by other UbiD family members. Here, we show that the *Pseudomonas aeruginosa* virulence attenuation factor PA0254*/*HudA is a pyrrole-2-carboxylic acid decarboxylase. The crystal structure of the enzyme in the presence of the reversible inhibitor imidazole reveals a covalent prFMN*–*imidazole adduct is formed. Substrate screening reveals HudA and selected active site variants can accept a modest range of heteroaromatic compounds, including thiophene-2-carboxylic acid. Together with computational studies, our data suggests prFMN covalent catalysis occurs via electrophilic aromatic substitution and links HudA activity with the inhibitory effects of pyrrole-2-carboxylic acid on *P. aeruginosa* quorum sensing.

## Introduction

**Figure 1:**
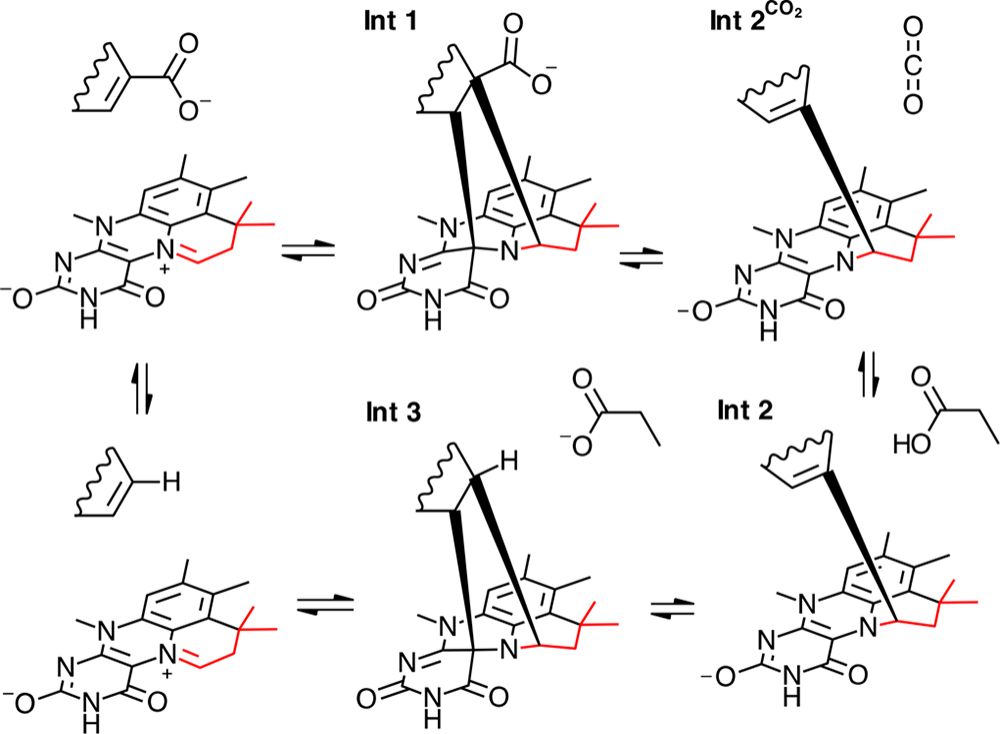
Covalent catalysis by prFMN in UbiD enzymes. prFMN cofactor is formed through FMN prenylation followed by oxidative maturation (prenylation shown in red). Unsaturated carboxylic acid is proposed to form a covalent adduct with the prFMN^iminium^ cofactor leading to decarboxylation. In the case of acrylic acid substrates, substantial evidence supports a 1,3-dipolar cycloaddition process leading to intermediate 1 (**Int 1**), followed by decarboxylation to form **Int 2 + CO**_**2**_. Exchange of CO_2_ with a conserved acidic residue leads to protonation to form **Int 3**, which is proposed to form product through cycloelimination. The exact nature of the various intermediate species remains unclear in the case of (hetero)aromatic substrates.

The UbiD family of enzymes catalyzes the reversible nonoxidative decarboxylation of a wide range of unsaturated aliphatic and aromatic compounds, the latter including phenolic compounds,^1^ heteroaromatics,^2^ phthalates,^3^ polycyclics,^4^ as well as benzene itself^5^ (recently reviewed in ref (6)). Recent insights into the UbiD mode of action came from studies on the fungal enzyme ferulic acid decarboxylase Fdc1.^7^ These revealed that UbiD enzymes require a modified flavin cofactor, prenylated-FMN (prFMN), for activity^8^ (Figure 1). The genetically associated UbiX acts as the flavin prenyltransferase, attaching a prenyl moiety to the N5 and C6 positions of reduced FMN, thereby extending the cofactor with a fourth nonaromatic ring.^9,10^ Following UbiD binding, the reduced prFMNH_2_ produced by UbiX is proposed to undergo oxidative maturation to yield the active prFMN^iminium^ species. The azomethine ylide character of the prFMN^iminium^ supports a reversible 1,3-dipolar cycloaddition underpinning the (de)carboxylase mechanism of Fdc1^8^ (Figure 1). Recent structural insights into a range of covalently bound substrate/cofactor adducts confirmed that cycloadducts are formed during the catalytic cycle.^11^ However, the extent to which 1,3-dipolar cycloaddition occurs for UbiD enzymes acting on (hetero)aromatic substrates has been questioned, as the necessary dearomatization of the substrate presents a substantial barrier to cycloadduct formation.

Unfortunately, recent structural and biochemical characterization of three UbiD decarboxylases acting on aromatic substrates has not yielded detailed mechanistic insights to answer this question. In the case of the canonical *Escherichia coli* UbiD, the purified enzyme failed to mature the active form of the cofactor, instead accumulating an inactive prFMN^radical^ species.^12^ Active enzymes could be obtained for HmfF (catalyzing the decarboxylation of 2,5-furandicarboxylic acid^13^) and AroY (decarboxylating protocatechuate to catechol^14^). In each case, crystals of the *holo*-enzyme did not yield any substrate complexes despite several attempts. The discrepancy between the closed, solvent-occluded conformation of the Fdc1 active site (which readily binds substrates or inhibitors) and the more open, solvent-accessible conformations (which hitherto have not yielded any ligand-bound structures) observed for the UbiD, HmfF, and AroY structures can be explained by a putative hinge motion of the prFMN-binding domain.^14−16^ Such a conformational change would link the postulated open and closed conformations, but as yet no UbiD enzyme has been demonstrated to exhibit both conformations. To further complicate matters, the dimeric Fdc1 belongs to a distinct branch of the UbiD family tree as compared to the hexameric UbiD/HmfF and AroY enzymes, reflected in the distinct substrate specificities of these enzymes. However, recent protein engineering studies on Fdc1 have been able to extend the substrate scope to include (hetero)aromatic compounds using a single active site substitution.^15^ This suggests that quaternary structure or position within the UbiD-family tree does not have a fundamental link to substrate specificity.

We sought to provide further detailed insights into the UbiD reaction with heteroaromatic substrates, focusing on pyrrole-2-carboxylate (P2C) decarboxylases. We here report that the UbiD-like *Pseudomonas aeruginosa* virulence attenuation factor PA0254/HudA is a close homologue of *Bacillus megaterium* PYR2910 pyrrole-2-carboxylate (P2C) decarboxylase and show that PA0254 is capable of prFMN-dependent decarboxylation of P2C as well as carboxylation of pyrrole. Structure determination of the PA0254 *holo*-enzyme reveals a closed conformation with a buffer-derived imidazole moiety covalently bound to the prFMN. Substrate screening reveals that a modest range of heteroaromatic substrates is accepted, including weak activity with thiophene-2-carboxylate. Structure-based semirational engineering supported a modest improvement in yields with the latter compound. In combination with the DFT computational studies, our data suggests that covalent catalysis occurs via electrophilic aromatic substitution.

## Experimental Procedures

### Cloning of PA0254 Pyrrole-2-carboxylate Decarboxylase

The gene encoding PA0254 (NC_002516) was codon optimized to remove codons that were rare in *E. coli* and synthesized (Genscript). The gene was amplified using Phusion polymerase (NEB) using the primers P0254pNIC28F (TACTTCCAATCCATGAACCGCTCGGCACTG) and P0254pNIC28R (GTGCGGCCGCAAGCTTCAGGCATCACCAAAGCC), and the PCR product was cloned into the *Nco*I and *Hind*III sites of pNic28-Bsa4 expression plasmid (Addgene) using Infusion HD (Clontech) to reproduce the construct reported by Jacewicz et al.^16^ Once the sequence of the desired insert was confirmed, the corresponding purified plasmid was transformed into *E. coli* BL21(DE3) with ubiXpET21b to provide sufficient levels of prFMN in vivo.^8^ BL21(DE3) cells were also transformed with PA0254pNic28 without ubiXpET21b.

### Mutagenesis

Mutagenesis primers were designed using the QuikChange Primer Design Program (http://www.genomics.agilent.com/primerDesignProgram.jsp). PCR was performed using Phusion polymerase (NEB). Template was removed by *Dpn*I (NEB) digest, and the PCR product was transformed into *E. coli* NEB5α. Once the presence of the desired mutation was confirmed by DNA sequencing, the plasmid was cotransformed with ubiXpET21b into *E. coli* BL21(DE3).

### Protein Expression

The enzymes were expressed in BL21(DE3) grown at 37 °C/180 rpm in TB broth supplemented with 50 μg/mL kanamycin and 50 μg/mL ampicillin. At mid log phase cells were induced with 0.25 mM IPTG and supplemented with 1 mM MnCl_2_, grown overnight at 17 °C/180 rpm, and then harvested by centrifugation (4 °C, 7000*g* for 10 min).

### Purification of His-Tagged Proteins

Cell pellets were resuspended in buffer A (200 mM KCl, 1 mM MnCl_2_, 50 mM Tris pH 7.5) supplemented with DNase, RNase, lysozyme (Sigma), and Complete EDTA-free protease inhibitor cocktail (Roche). Cells were lysed using a French press at 20 000 psi, and the lysate was clarified by centrifugation at 125 000*g* for 90 min. The supernatant was applied to a Ni–NTA agarose column (Qiagen). Initially, imidazole was used to elute the protein with the column being washed with 3 column volumes of buffer A supplemented with 10 mM imidazole and protein eluted in 1 mL fractions with buffer A supplemented with 250 mM imidazole. Once imidazole was observed to be binding to the cofactor, subsequent purifications utilized histidine to elute the protein, with the column being washed with 3 column volumes of buffer A supplemented with 10 mM histidine and protein eluted in 1 mL fractions with buffer A supplemented with 100 mM histidine. Samples were subjected to SDS-PAGE analysis, and fractions found to contain the purified protein were pooled. Imidazole/histidine was removed using a 10-DG desalting column (Bio-Rad) equilibrated with buffer A. Protein was aliquoted and flash frozen until required. Cells grown for the production of *apo* PA0254 (i.e., without ubiXpET21b) were resuspended in 200 mM NaCl, 50 mM Tris-HCl, pH 7.5 or 200 mM KCl, 50 mM Tris-HCl, pH 7.5. Other aspects of the purification remained constant with *holo* preparations using imidazole as the eluent.

### UV–vis Spectroscopy/Protein Quantification and Decarboxylation Assays

UV–vis absorbance spectra were recorded with a Cary 50 Bio UV–Vis spectrophotometer (Varian). The protein concentration was estimated from the *A*_280_ absorption peak with extinction coefficients calculated from the primary amino acid sequence using the ProtParam program on the ExPASy proteomics server. PA0254 concentration was estimated using ε_280_ = 78 380 M^–1^ cm^–1^. Initial rates of pyrrole-2-carboxylate (P2C) decarboxylation were determined by monitoring P2C concentration by UV–vis spectroscopy at 255 nm using an extinction coefficient ε_255_ = 18 000 M^–1^ cm^–1^. Assays were performed with various concentrations of substrate dissolved in 350 μL of 50 mM KCl, 50 mM NaPi, pH 6 using a 1 mm path length cuvette.

### PA0254^UbiX^ Decarboxylation Reactions Assayed by HPLC

Typical assays containing 10 mM pyrrole-2-carboxylate, 50 mM KCl, and 50 mM NaPi, pH 6, were incubated with and without enzyme at 30 °C overnight. The sample was centrifuged at 16 100*g* to remove precipitate, and 50 μL was added to 450 μL of 50% v/v H_2_O/acetonitrile. Sample analysis was performed using an Agilent 1260 Infinity Series HPLC equipped with a UV detector. The stationary phase was a Kinetex 5 μm C18 100A column, 250 × 4.6 mm. The mobile phase was acetonitrile/water (50/50) with 0.1% TFA at a flow rate of 1 mL/min, and unless otherwise stated, detection was performed at a wavelength of 210 nm.

### PA0254^UbiX^ Carboxylation Reactions Assayed by HPLC

Typical assays containing 50 mM pyrrole, 100 mM KPi, pH6, and 1 M KHCO_3_ (final pH 7.5) were incubated with and without PA0254 enzyme at 30 °C overnight. The sample was centrifuged at 16 100*g* to remove the precipitate, and 20 μL was added to 980 μL of 50% v/v H_2_O/acetonitrile. Sample analysis was performed by HPLC as described above.

### PA0254^UbiX^ Activity Dependency on Metal Ions

To test for the metal ion requirements of PA0254^**UbiX**^, protein purified in the presence of NaCl or KCl was reconstituted with prFMN produced in vitro. Two UbiX reactions were performed in parallel, with 200 mM NaCl or KCl, 50 mM Tris, pH 7.5, as the diluent; 0.1 mM FMN, 0.5 mM DMAP, 0.5 mM NADH, 50 μM UbiX, and 2 μM Fre (*E. coli* NAD(P)H-flavin reductase) were mixed with each reaction inside a Belle Technologies Anaerobic chamber. The reactions were incubated for 3 h prior to separation of prFMNH_2_ from the proteins using a 10 kDa MWCO microcentrifugal concentrator (Sartorius). To a final concentration of 30 μM PA0254 in either NaCl or KCl buffer A, filtrate containing prFMN was added to a final concentration of 10 μM in the respective salt, with MnCl_2_ or MgCl_2_ added to a final concentration of 100 μM. Controls with no MnCl_2_/MgCl_2_ were performed as was a control with no additional MnCl_2_/MgCl_2_, and prFMN. P2C was dissolved in 50 mM NaCl, 50 mM NaPi, pH 6 or 50 mM KCl, 50 mM KPi, pH 6. PA0254 in the respective salt was added to a final concentration of 0.75 μM (final added [prFMN] = 0.25 μM). HPLC assays were performed as described above with the exception that reactions were allowed to proceed for 1 h at 25 °C prior to quenching with acetonitrile + 0.1% TFA in a 1:1 volume ratio with the reaction, followed by centrifugation and a 1 in 5 dilution with 50% (v/v) H_2_O/acetonitrile + 0.1% TFA. Detection and depletion of P2C was performed at 270 nm.

### ^1^H NMR Monitored Enzyme-Catalyzed Deuterium Exchange

Ten millimolar substrate, 100 mM NaPi, pH 5.6, in D_2_O was incubated overnight at 30 °C with and without 5 μM PA0254^UbiX^. Data were collected on a Bruker 500 MHz NMR spectrometer and QCI-F cryoprobe at 298 K with a 4 min accumulation time.

### EPR Spectroscopy

Continuous-wave X-band (∼9.4 GHz) EPR spectra were recorded with a Bruker E500/580 EPR spectrometer with a Bruker “Super High Q” cavity (ER 4122SHQE) coupled to an Oxford Instruments ESR900 helium flow cryostat for temperature control. Spectra were collected at 20 K using 10 μW microwave power, 100 kHz field modulation frequency, and 1 G modulation amplitude.

### Crystallization of PA0254^UbiX^

Purified PA0254^UbiX^ in 200 mM NaCl, 50 mM Tris, pH 7.5, was concentrated in a Vivaspin 30 kDa MWCO spin concentrator to a final concentration of ∼10–20 mg/mL. Initial screening by sitting drop was performed; mixing 0.3 μL of protein with 0.3 μL of mother liquor led to crystals in a variety of conditions when incubated at 21 °C. The best-performing crystals originated from well D3 of the Morpheus commercial screen (Molecular Dimensions) consisting of 0.12 M alcohols, 0.1 M imidazole/MES, pH 6.5, 20% v/v glycerol, and 10% w/v PEG 4000. Crystals of PA0254^UbiX^ N318H mutant were attained as above but were grown in condition H7 of the Morpheus screen (Molecular Dimensions) consisting of 0.1 M amino acids, 0.1 M NaHEPES/MOPS buffer, pH 7.5, 20% v/v glycerol, and 10% w/v PEG 4000.

### Diffraction Data Collection and Structure Elucidation

Crystals were flash cooled in liquid nitrogen. Data were collected at Diamond beamlines and subsequently handled using the CCP4 suite.^17^ All data were reduced and scaled using XDS.^18^ Interpretable maps were obtained following molecular replacement with the available *apo*-PA0254 crystal structure (PDB code 4IP2). The initial model was iteratively rebuilt and refined using Coot and REFMAC5.^17^ The final model was refined using data extending to 1.65 Å and contains 4 monomers in the asymmetric unit. For final data collection and refinement statistics, see Table 1.

**Table 1:** Crystallographic Data Collection and Refinement Parameters

	PAO254/HudA imidazole complex	PAO254/HudA N318A FMN complex
PDB	7ABN	7ABO
resolution range (Å)	50–1.65 (1.68–1.65)	47.0–1.95 (1.98–1.95)
space group	*P*12_1_ 1	*P*12_1_ 1
unit cell	107.81, 55.48, 199.53, 90, 99.93, 90	107.88, 55.57, 198.99, 90, 100.0, 90
unique reflections	27 9939 (18 328)	16 8873 (8399)
multiplicity	3.1 (2.9)	3.2 (3.0)
completeness (%)	96.4 (86.0)	99.2 (99.1)
mean *I*/sigma(*I*)	8.6 (1.5)	11.7 (1.4)
*R* meas	0.093 (0.812)	0.081 (1.025)
CC1/2	1.0 (0.7)	1.0 (0.5)
*R* work	0.201 (0.298)	0.214 (0.327)
*R* free	0.235 (0.341)	0.246 (0.354)
RMS bonds (Å)	0.022	0.004
RMS angles (deg)	2.12	0.68
Ramachandran favored (%)	96.3	95.9
Ramachandran allowed (%)	3.4	3.7
Ramachandran outliers (%)	0.3	0.4
average *B* factor (Å^2^)	19.0	38.0

### DFT Calculations

DFT calculations of the prFMN–substrate adducts were performed using Gaussian 09, revision D.01 at the B3LYP/6-311++G(d,p) level of theory with the D3 version of Grimme’s dispersion with Becke–Johnson damping^19^ and a generic polarizable continuum model of water. Additional details of the models are provided in the Supporting Information.

## Results

### Pyrrole-2-carboxylate Decarboxylase Belongs to the HudA Clade

**Figure 2:**
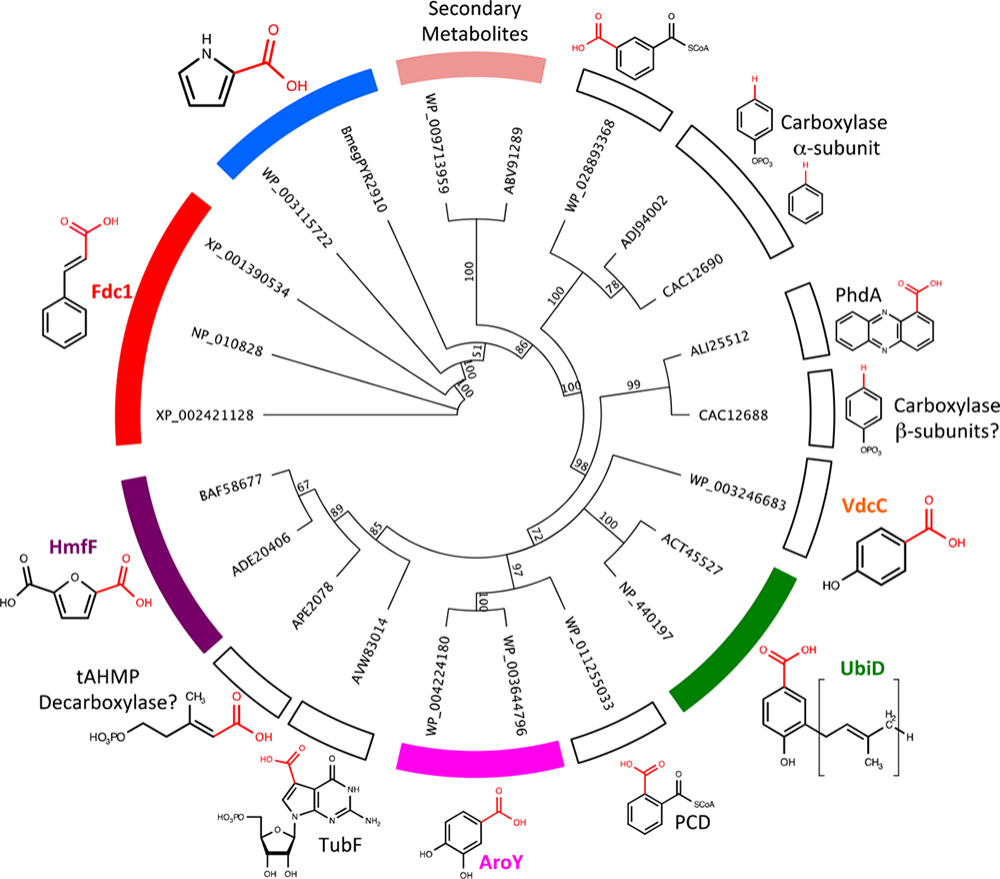
Phylogenetic analysis of known UbiD family. *B. megaterium* PYR2910 P2C decarboxylase, *A. niger* Fdc1 (XP_001390534),^22^*Saccharomyces cerevisiae* Fdc1 (NP_010828),^23^*E. coli* UbiD (ACT45527),^24^*Synechocystis* sp. PCC 6803 4-hydroxy-3-solanesylbenzoate decarboxylase (NP_440197),^25^*P. aeruginosa* PA0254/HudA (WP_003115722),^16^*Streptomyces griseochromogenes* TtnD (ABV91289),^26^*Streptomyces himastatinicus* SmdK (WP_009713959),^27^*C. basilensis* HmfF (ADE20406),^20^*Bacillus subtilis* VdcC (WP_003246683),^28^*Thauera aromatica* phenylphosphate carboxylase alpha (CAC12690) and beta (CAC12688) subunits,^29^*Clostridium* BF benzene carboxylase (ADJ94002),^30^*Klebsiella pneumoniae* AroY (WP_004224180),^31^*Lactobacillus plantarum* 3,4-dihydroxybenzoate decarboxylase LpdC (WP_003644796),^32^*Aromatoleum aromaticum* phthalyl-CoA decarboxylase (WP_011255033),^33^*Syntrophorhabdus aromaticivorans* isophthalyl-CoA decarboxylase (WP_028893368),^34^*Streptomyces tubercidicus* TubF (AVW83014),^35^ and *Aeropyrum pernix* (APE_2078).^36^ Different branches can be grouped by substrate specificity. Structures of substrates are shown with the leaving group (carboxylate for decarboxylases, hydrogen for carboxylases) in red. Colored segments indicate subfamilies for which crystal structures are available. Sequences of UbiD homologues were aligned using T-coffee, and Trees were generated using Geneious Tree builder using the neighbor-joining method.

The amino acid sequence of the *B. megaterium* PYR2910 pyrrole-2-carboxylate decarboxylase was kindly provided by Professor Yoshida of Gifu University. Phylogenetic analysis of the PYR2910 sequence indicates that it clusters with UbiD clades containing the fungal Fdc1 enzymes and *Streptomyces* decarboxylase genes. The latter are involved in secondary metabolite biosynthesis, and both enzymes are typically associated with acrylic acid-type substrates (Figure 2). Despite the distinct heteroaromatic substrate specificity, the *B. megaterium* PYR2910 P2C decarboxylase was found to possess 40% identity with *Aspergillus niger* Fdc1, as opposed to 31% identity with the *Cupriavidus basilensis* HmfF furan-2,5-dicarboxylic acid decarboxylase.^13,20^ The nearest homologue that has been studied in detail is PA0254 from *P. aeruginosa* PAO1 (44% identity).^16^ Indeed, the first UbiD crystal structure to be reported was that of *P. aeruginosa* PA0254.^16^ However, the precise function or activity of PA0254 (also known as HudA) was unknown, although it has been implicated as a virulence attenuation factor.^21^ As a consequence, the structure obtained was that of the *apo*-enzyme, lacking the prFMN cofactor that had yet to be identified at the time. Furthermore, the *B. megaterium* PYR2910 enzyme,^2^ PA0254, and the fungal Fdc1 form dimers,^8,16^ in contrast to the hexameric (hetero)aromatic (de)carboxylases UbiD,^12^ HmfF ^13^, and AroY.^14^ This confirms that a quaternary structure or position within the UbiD family tree does not always correlate with substrate specificity.

### PA0254 Is a Pyrrole-2-carboxylate Decarboxylase

**Figure 3:**
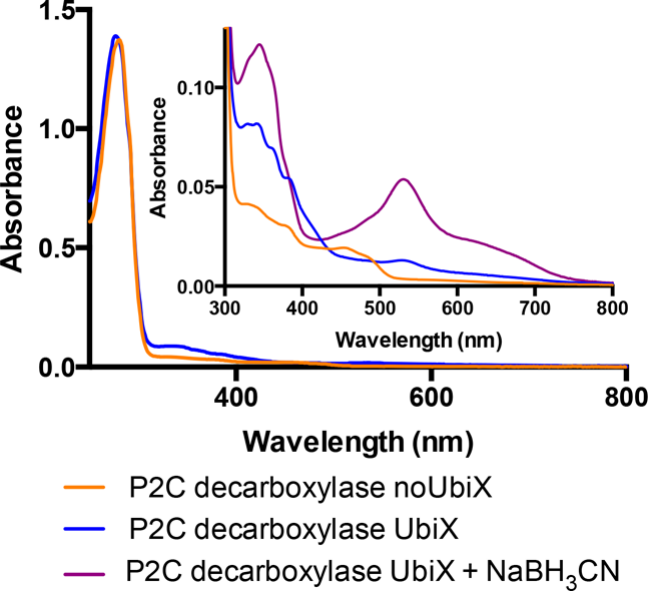
UV–vis spectrum of PA0254. PA0254 expressed with (blue) and without (yellow) UbiX and after treatment with NaBH_3_CN under aerobic conditions (purple). Spectra were normalized with respect to the A_280_ peak. (Inset) Close up of the cofactor-related spectral features in the 300–800 nm range.

We expressed His-tagged PA0254 with and without UbiX in *E. coli*. A comparison of the UV–vis spectra of the purified proteins reveals UbiX coexpression has a drastic effect on the PA0254 properties (Figure 3). In the presence of UbiX (denoted as PA0254^UbiX^), spectral features associated with prFMN are observed, while in the absence of UbiX coexpression, a more flavin-like spectrum is observed. For the PA0254^UbiX^ sample, the presence of a minor 550 nm feature is indicative of the presence of the prFMN^radical^ semiquinone species.^8^ Addition of NaBH_3_CN to PA0254^UbiX^ resulted in the development of a visible purple color under aerobic conditions with a corresponding increase in the *A*_550_ peak. This is consistent with conversion of the major prFMN^iminium^ species to the prFMN^radical^ as previously reported for *A. niger* Fdc1.^8^

**Figure 4:**
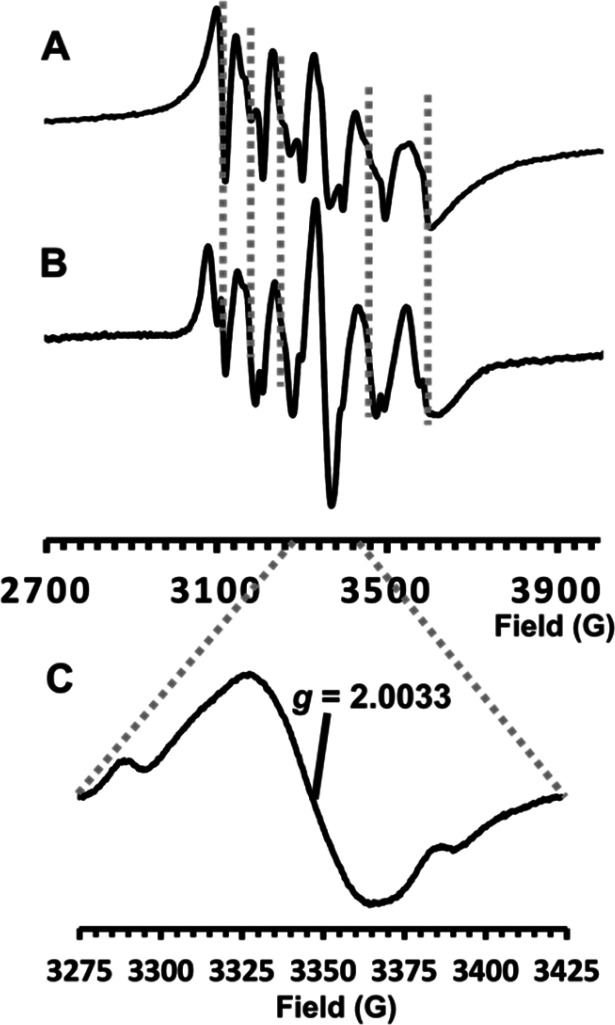
X-band CW EPR spectra of PA0254^UbiX^. EPR spectra of (A) Mn^2+^ in aqueous buffer solution and (B) PA0254^UbiX^, both recorded at 20 K using 0.5 mW microwave power and a modulation amplitude of 7 G. (C) *g* = 2 region of the PA0254^UbiX^ spectrum recorded at 20 K using 10 μW microwave power and 1 G modulation amplitude.

The prFMN^radical^ so formed is detectable in the X band CW EPR spectrum (Figure 4) at the center of the six line pattern arising from the *m*_s_ = ±1/2 manifold of the *S* = 5/2 Mn^2+^ ion, the six lines arising from hyperfine interaction with the *I* = 5/2 ^55^Mn nucleus. Comparison of Figure 4B with spectrum Figure 4A demonstrates that this is protein-bound, not free, Mn^2+^, and Figure 4C presents the radical signal recorded under low power and modulation amplitude to show that it has the same form as the prFMN_radical_: Mn^2+^-coupled signal of *A. niger* Fdc1 (8) and *E. coli* UbiD (12).

**Figure 5:**
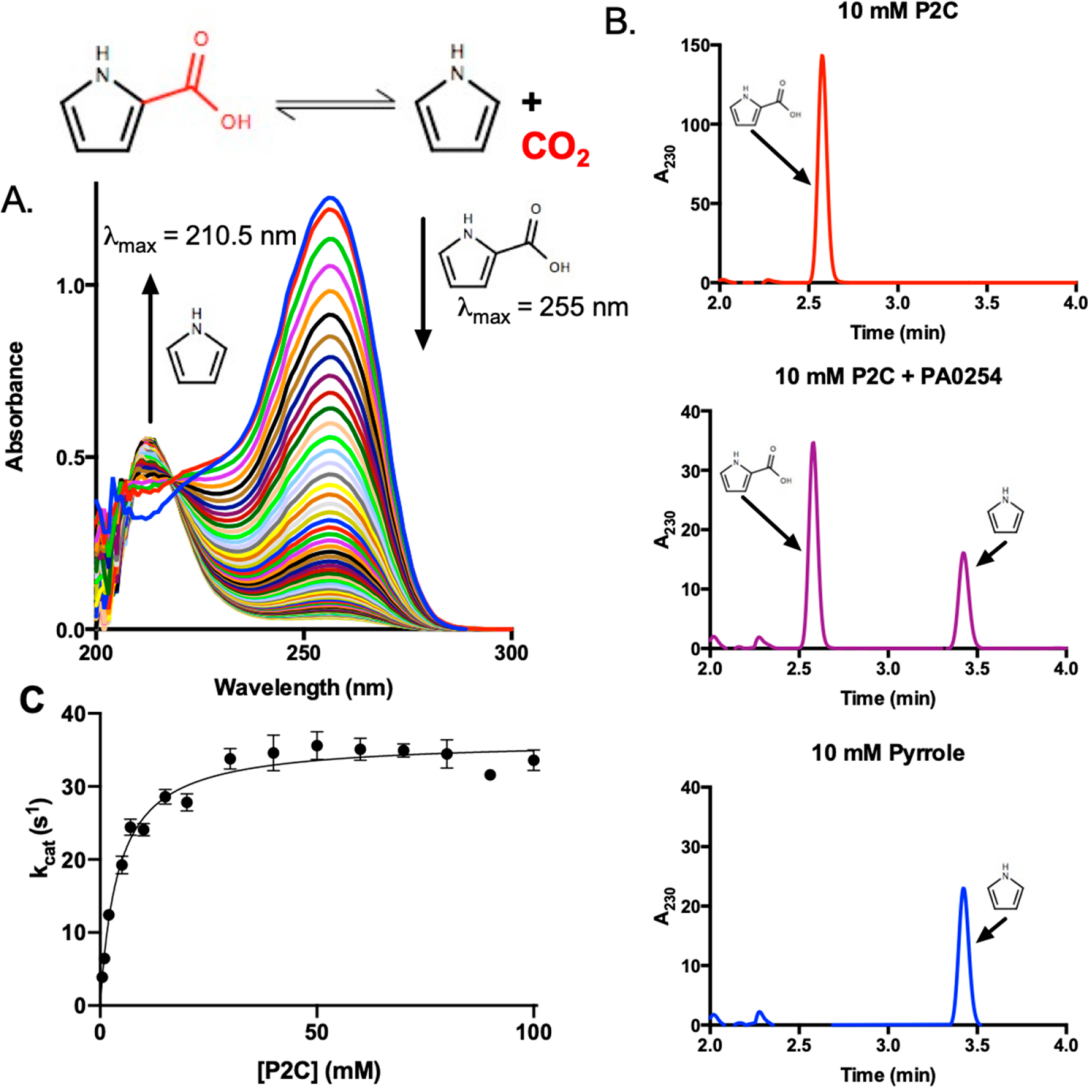
Pyrrole-2-carboxylate (P2C) decarboxylase activity of PA0254. (A) UV–vis spectra of P2C following addition of PA0254, revealing a peak corresponding to pyrrole production (210.5 nm) appears over time. (B) HPLC analysis of P2C incubated with PA0254 confirms formation of a product with the same retention time as pyrrole. (C) Michaelis–Menten kinetics of P2C decarboxylation.

As previously reported by Jacewicz et al., no decarboxylation activity could be detected with the Fdc1 substrate cinnamic acid. However, when PA0254^UbiX^ was added to pyrrole-2-carboxylate, pyrrole was readily formed as confirmed by UV–vis (Figure 5A) and HPLC analysis (Figure 5B). In contrast, PA0254 (i.e., expressed in the absence of UbiX) possessed little or no activity, confirming prFMN is required for P2C decarboxylation. The Michaelis–Menten kinetics for P2C decarboxylation indicated *K*_m_^app^ and *k*_cat_^app^ values of 4.3 (±0.5) mM and 35.8 (±0.8) s^–1^, respectively (Figure 5C), values similar to those previously reported for the *B. megaterium* homologue.^2^ These are reported as apparent values in view of the presence of a minor population of inactive prFMN radical species complicating accurate quantification of active enzyme concentration. However, the relative increase in the prFMN radical signal following NaBH_3_CN reduction under aerobic conditions suggests the signal in the as-isolated samples accounts for less than 10% of the total population.

### PA0254^UbiX^ Activity Is Light Sensitive

**Figure 6:**
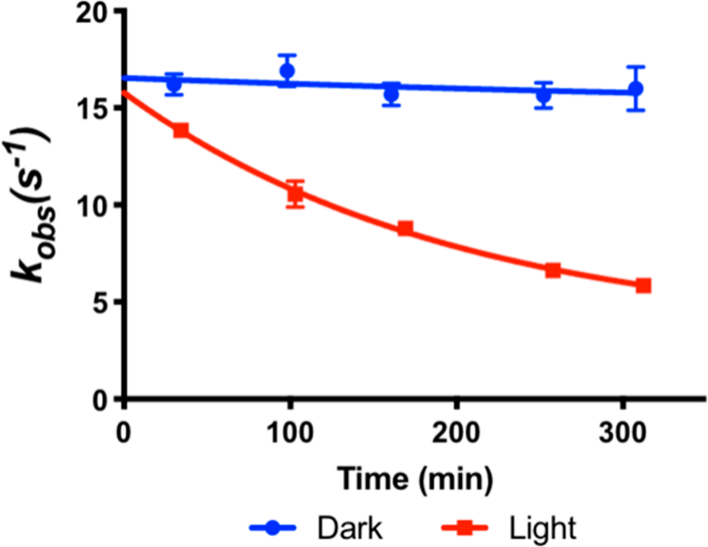
PA0254 activity is light sensitive. PA0254 activity under aerobic conditions in the light (red) and in the dark (blue). Assays were performed against 1 mM P2C. Error bars represent SEM, *n* = 3.

Previous reports indicated that the activity of the *B. megaterium* enzyme was oxygen sensitive, with the addition of reducing reagents required to stabilize the enzyme.^37^ Fdc1 has also been reported to lose activity over time,^8^ in this case the result of light-induced isomerization and inactivation of prFMN^iminium^.^38^ Similar to Fdc1, PA0254^UbiX^ was found to lose activity with a half-life of ∼140 min when incubated on ice in a transparent tube. By contrast, enzyme activity was found to be stable when kept in the dark (Figure 6).

### PA0254^UbiX^ Catalyzes Pyrrole H/D Exchange

**Figure 7:**
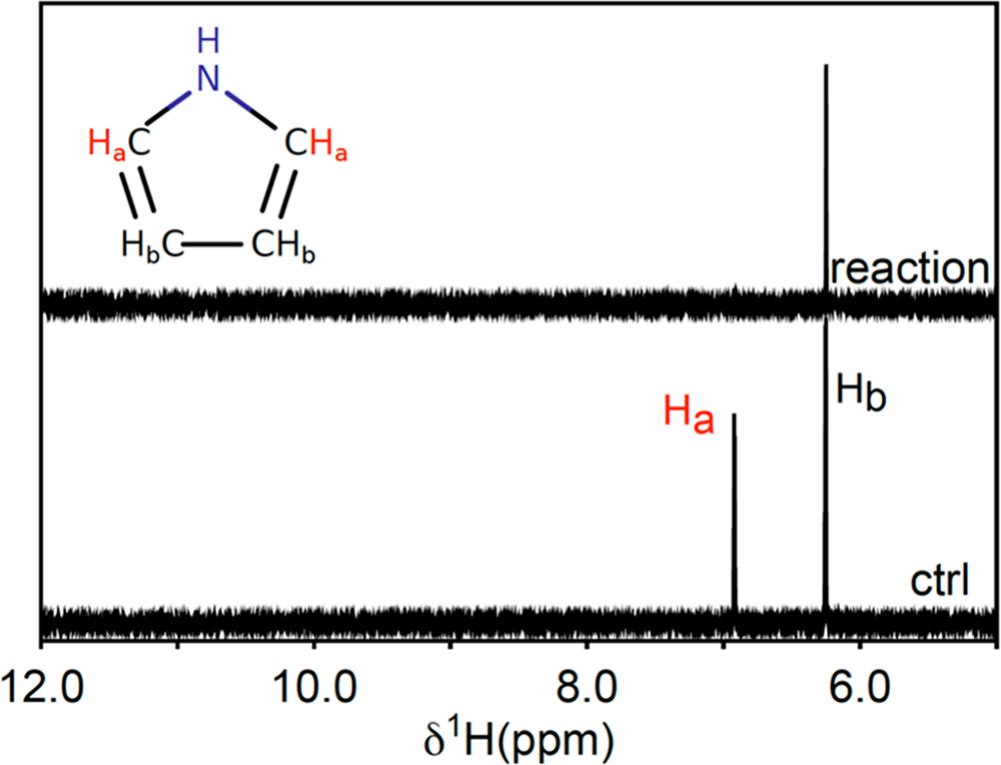
^1^H NMR analysis of pyrrole H/D exchange. ^1^H NMR spectra of pyrrole in D_2_O when incubated with (reaction) and without (ctrl) PA0254^UbiX^.

Previous work has shown that UbiD decarboxylases are capable of catalyzing deuterium exchange on the decarboxylation reaction products.^39^^1^H NMR showed that incubation of pyrrole with PA0254^UbiX^ in D_2_O resulted in depletion of the resonance peak at 6.9 ppm, consistent with exchange of both protons in positions 2 and 5 (denoted H_a_) with deuterons (Figure 7).

### PA0254^UbiX^ Catalyzes Pyrrole Carboxylation at Elevated [CO_2_]

**Figure 8:**
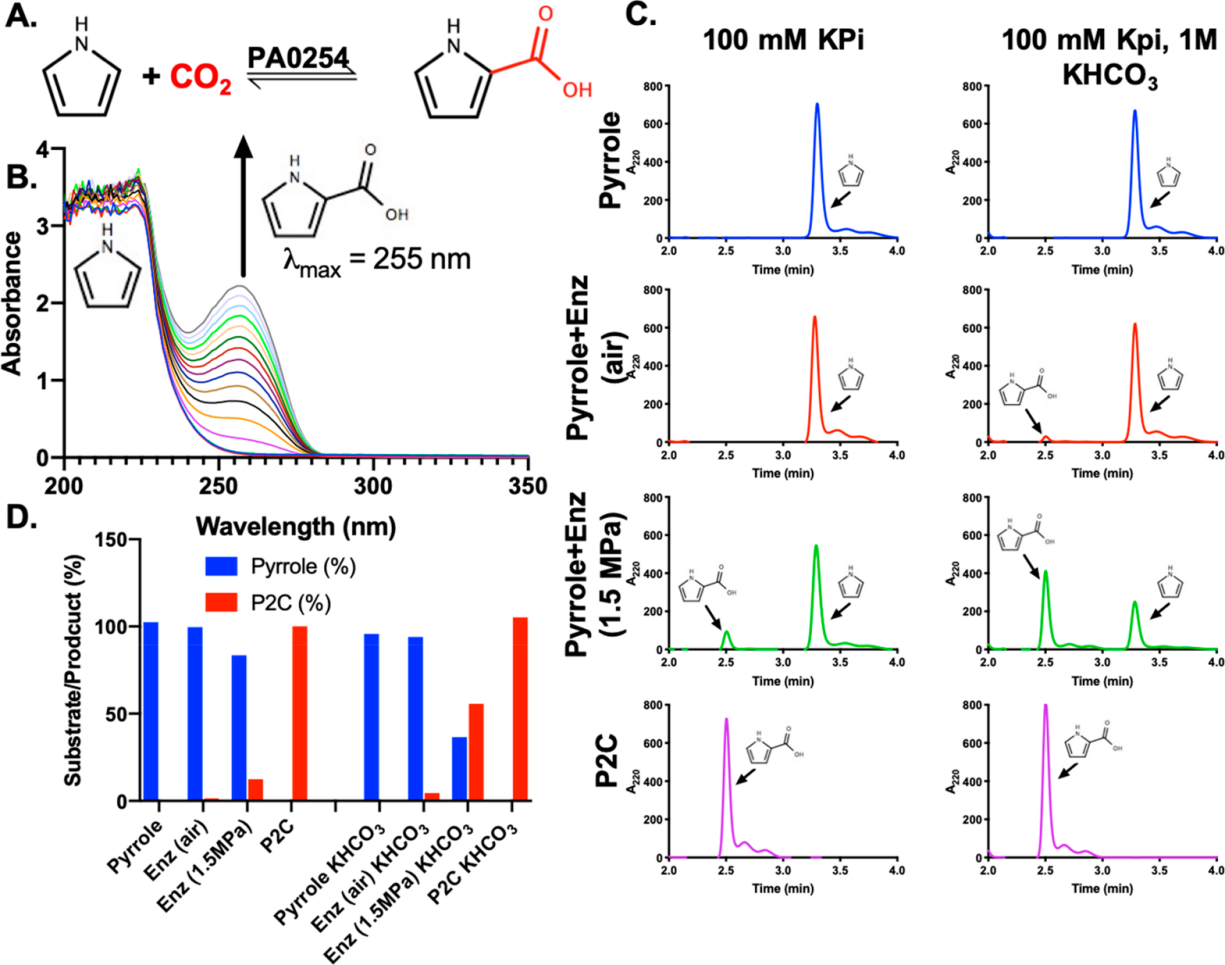
PA0254^UbiX^ catalyzed carboxylation of pyrrole to P2C. (A) Schematic of pyrrole carboxylation. (B) UV–vis spectrum of pyrrole following addition of PA0254^UbiX^ in the presence of KHCO_3_. Spectra recorded at 1 min intervals following addition of PA0254^UbiX^. Peak at 255 nm increases in intensity over time corresponding to P2C production. (C) HPLC analysis of pyrrole incubated with PA0254 in the presence of KHCO_3_ and/or pressurized CO_2_, confirming that the product has the same retention time as P2C. (D) Relative conversions of pyrrole to P2C under the conditions shown in C. Highest proportion of pyrrole converted to P2C was 55% in the presence of KHCO_3_ and 1.5 MPa pressurized CO_2_.

*B. megaterium* PYR2910 was previously reported to catalyze the carboxylation of pyrrole in the presence of elevated concentrations of CO_2_, either through CO_2_ at high pressure or via addition of high concentrations of bicarbonate. To investigate the ability of PA0254^UbiX^ to catalyze the carboxylation of pyrrole, the enzyme was incubated with 25 mM pyrrole and 0.5 M potassium bicarbonate. UV–vis spectra recorded at 1 min intervals after addition of PA0254^UbiX^ revealed that a peak centered at 255 nm appeared and increased over time, consistent with the production of P2C with a *k*_obs_ of 4.6 s^–1^ (Figure 8). HPLC analysis of reactions incubated overnight with 50 mM pyrrole and 1 M potassium bicarbonate and/or under pressurized CO_2_ (1.5 MPa) revealed a peak with a retention time of 2.5 min that comigrates with a P2C standard (Figure 8C). The highest proportion of pyrrole converted to P2C was in the presence of both 1 M KHCO_3_ and 1.5 MPa CO_2_ (Figure 8D), whereas no P2C could be detected in the absence of additional bicarbonate/CO_2_ or enzyme.

### PA0254^UbiX^ Substrate Specificity Is Restricted

**Figure 9:**
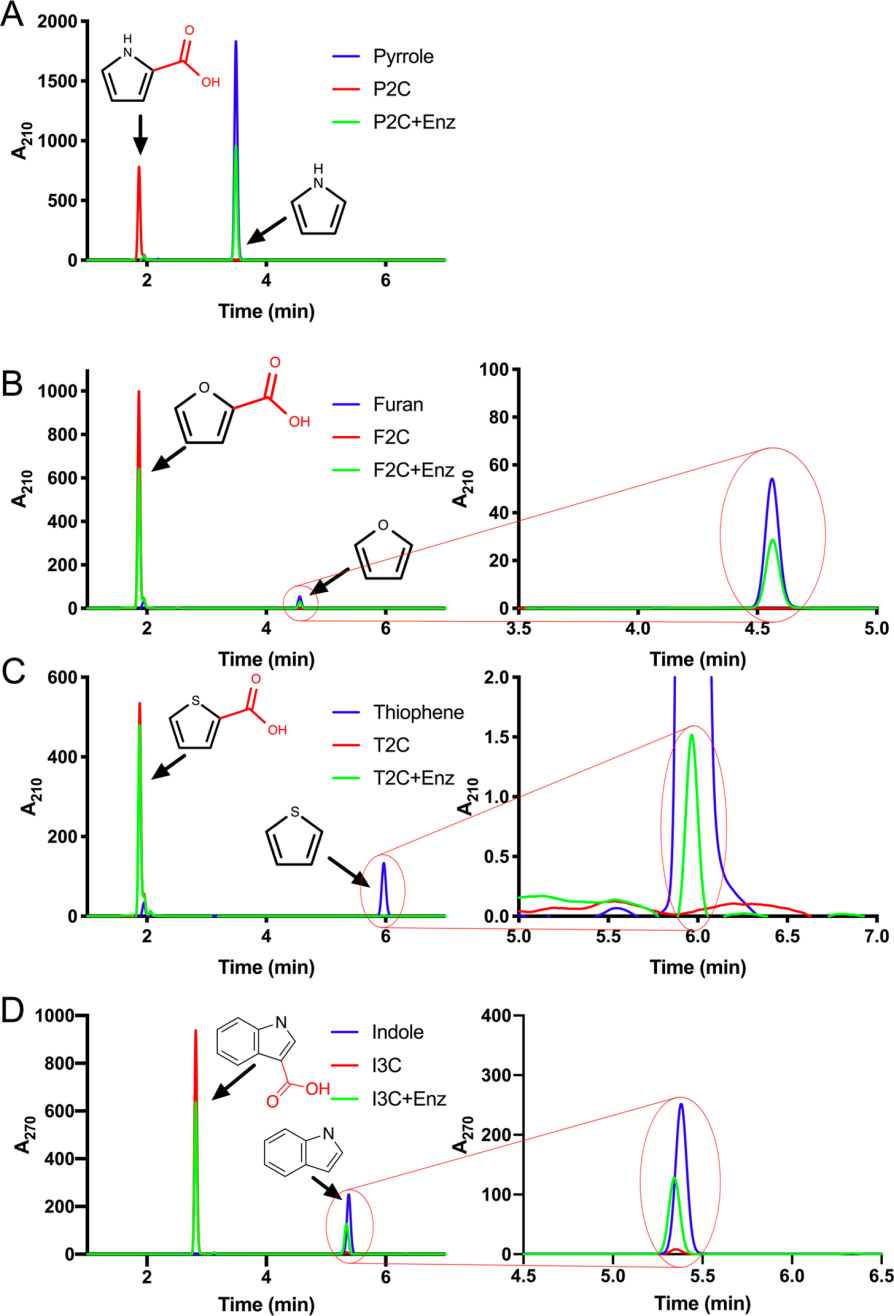
PA0254^UbiX^ substrate scope. Activity of PA0254^UbiX^ with heteroaromatic substrates including (A) pyrrole-2-carboxylate (P2C), (B) furan-2-carboxylate (F2C), (C) thiophene-2-carboxylate (T2C), and (D) indole-3-carboxylate (I3C). Ten millimolar product standards are shown in blue, while 10 mM substrate with and without enzyme are shown in green and red, respectively. Panels on the right show a zoom in of the adjacent chromatograms. See Figure 15C for a comparison of substrate depletion levels following addition of and incubation with PA0254^UbiX^ for a range of heteroaromatic acids.

**Figure 10:**
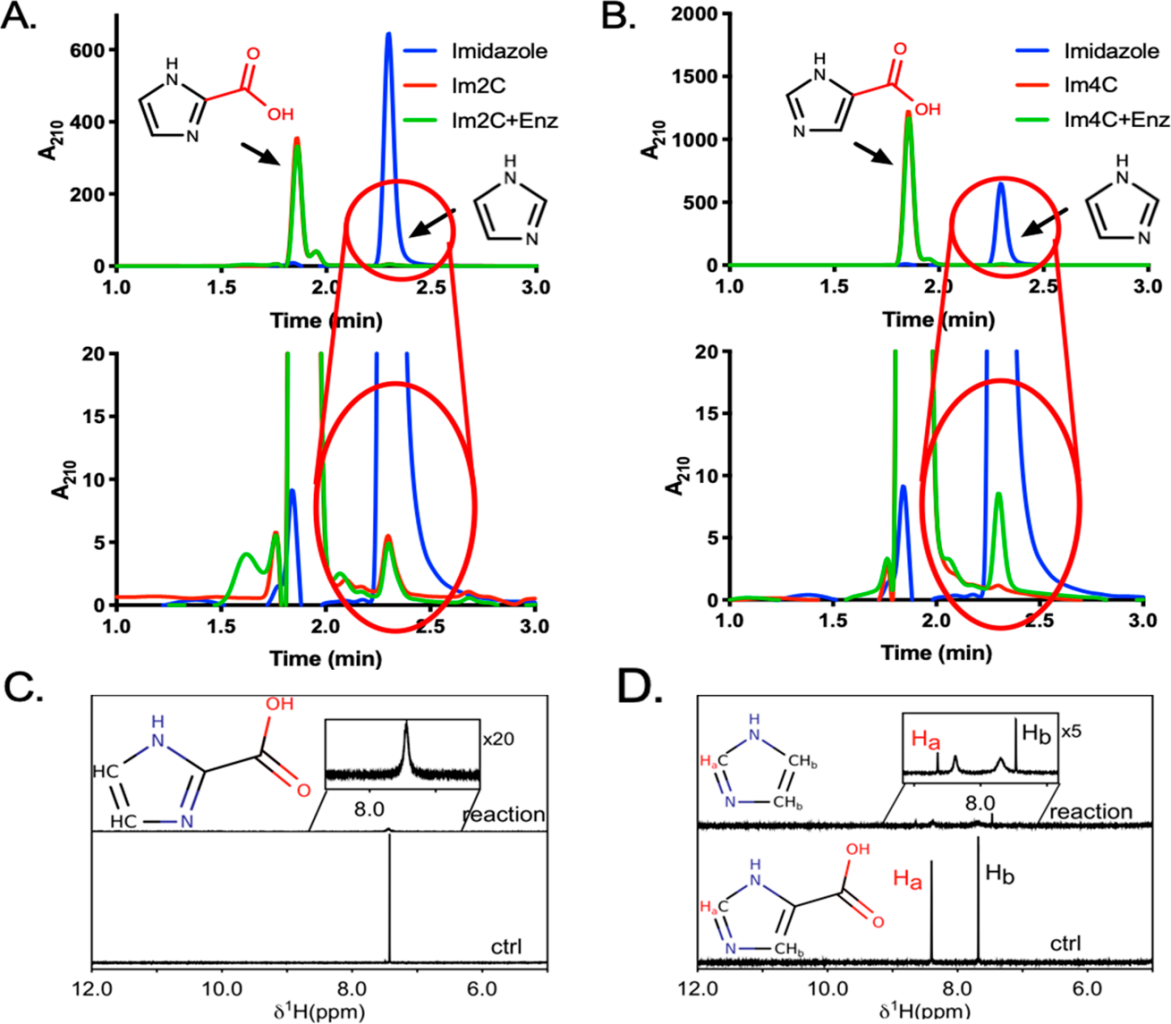
Activity of PA0254^UbiX^ with imidazole substrates. HPLC chromatograms of (A) imidazole-2-carboxylate (Im2C) and (B) imidazole-4-carboxylate (Im4C). Ten millimolar product standards are shown in blue, while 10 mM substrate with and without enzyme are shown in green and red, respectively. Lower panels show a zoom in to the above chromatograms; 0% of Im2C and 1.3% of Im4C is converted under the conditions tested. (C and D) ^1^H NMR analysis of Im2C (C) and Im4C (D) with (reaction) and without (ctrl) PA0254^UbiX^.

PA0254^UbiX^ activity was screened with various acids in order to determine the substrate range. Evidence of decarboxylase activity could be found with 3-methylpyrrole-2-carboxylate, indole-3-carboxylate, and furan-2-carboxylate with very low activity in the case of thiophene-2-carboxylate (Figure 9). In contrast, no evidence for the decarboxylation of pyrrole-3-carboxylate, indole-2-carboxylate, or benzoic acid was observed under the conditions tested. To test whether PA0254^UbiX^ could also decarboxylate imidazole-based compounds, we incubated the enzyme with imidazole-2-carboxylate (Im2C) and imidazole-4-carboxylate (Im4C). A small quantity of imidazole was produced in an enzyme-dependent manner from Im4C, while enzyme-dependent decarboxylation of Im2C was not observed (Figure 10). This was confirmed by monitoring the reaction using ^1^H NMR. In the presence of PA0254^UbiX^, the substrate-derived ^1^H peaks for both Im2C and Im4C broadened, possibly indicating binding to the enzyme. Two new peaks corresponding to imidazole formed only with Im4C (Figure 10C and 10D).

### PA0254^UbiX^ Crystal Structure Determination

**Figure 11:**
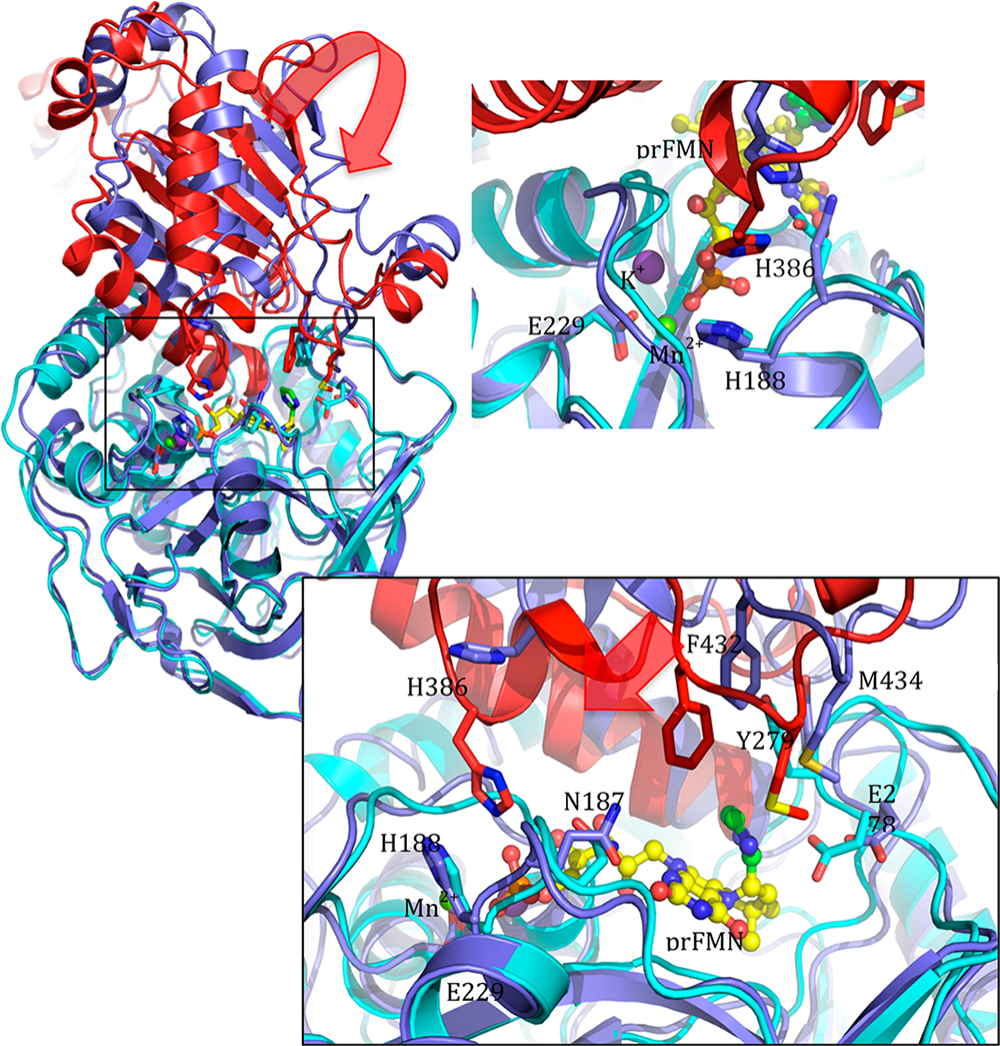
Cofactor binding in PA0254^UbiX^. Overlay of the *apo-*PA0254 structure (in blue) with the PA0254^UbiX^ holo-enzyme structure (in red/cyan) reveals the distinct position of the oligomerization domain with respect to the prFMN binding domain. Domain motion required to change from the *apo-*PA0254 conformation (in blue) to the *holo*-PA0254^UbiX^ (in red) leads to closure of the prFMN binding cleft, concomitant with active site closure. While the *apo-*PA0254 structure contains Mg^2+^, the *holo*-PA0254^UbiX^ contains Mn^2+^ and K^+^ ions that establish an ionic network with the prFMN phosphate group.

**Figure 12:**
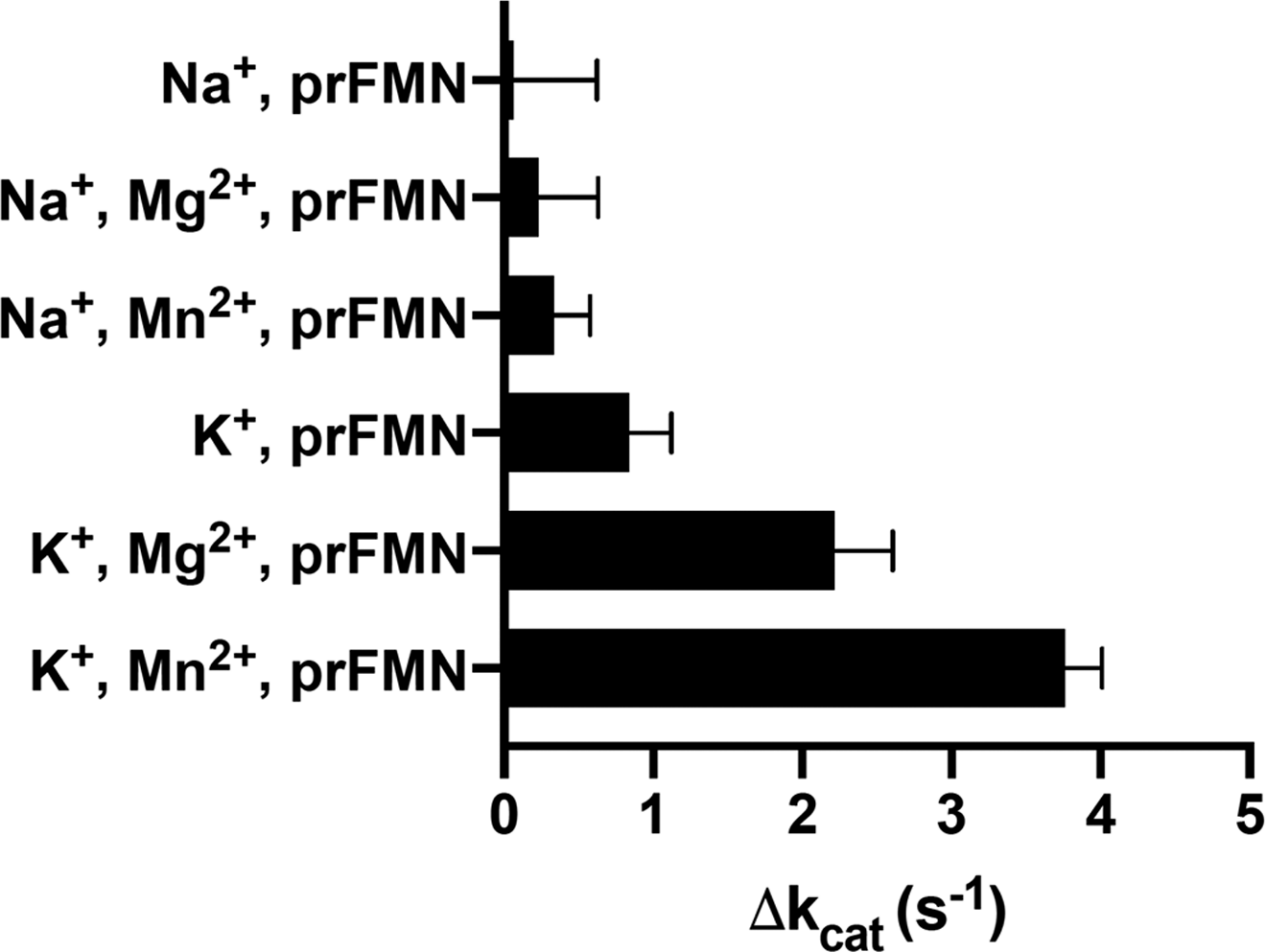
PA0254 activity is Mn^2+^and K^+^ dependent. Bar chart of *apo-*PA0254 P2C decarboxylation activity following reconstitution with either Na^+^/K^+^ and Mg^2+^/Mn^2+^ and prFMN. Activity was highest in the presence of both Mn^2+^ and K^+^ ions.

PA0254^UbiX^ was screened against 480 crystallization conditions and found to readily crystallize in a range of conditions. Unfortunately, several crystal forms suffered from various twinning pathologies, hindering structure determination. Crystals obtained in the presence of 100 mM imidazole (acting as the buffering agent) did occasionally yield nontwinned crystals which were used for data collection and refinement. A structure was obtained to 1.65 Å resolution using the *apo*-PA0254 structure as a molecular replacement model. The asymmetric unit (AU) contains two PA0254^UbiX^ dimers, with each monomer found to bind a prFMN cofactor via Mn^2+^ and K^+^ coordination (Figure 11). The identification of both ions is based on the electron density, the observation that PA0254^UbiX^ requires both Mn^2+^ and K^+^ for activity (Figure 12). The overall conformation and position of the prFMN binding domain is highly similar in the four monomers present in the AU and resembles the closed conformation previously observed for the *A. niger* Fdc1. Comparison with the previously reported *apo*-PA0254 structures reveals that considerable reorientation occurs in various loop regions associated with Mn^2+^ and/or prFMN binding.

Furthermore, the relative position of the prFMN binding domain with respect to the C-terminal dimerization domain is distinct in the *apo*-structures, adopting a more open conformation. This suggests that the open-to-closed transition is affected by prFMN and/or substrate binding. In the case of the related *A. niger* Fdc1, cofactor binding has indeed been demonstrated to affect the protein overall conformation using mass spectrometry.^40^

### PA0254^UbiX^ Crystal Structure Yields an Imidazole Adduct

**Figure 13:**
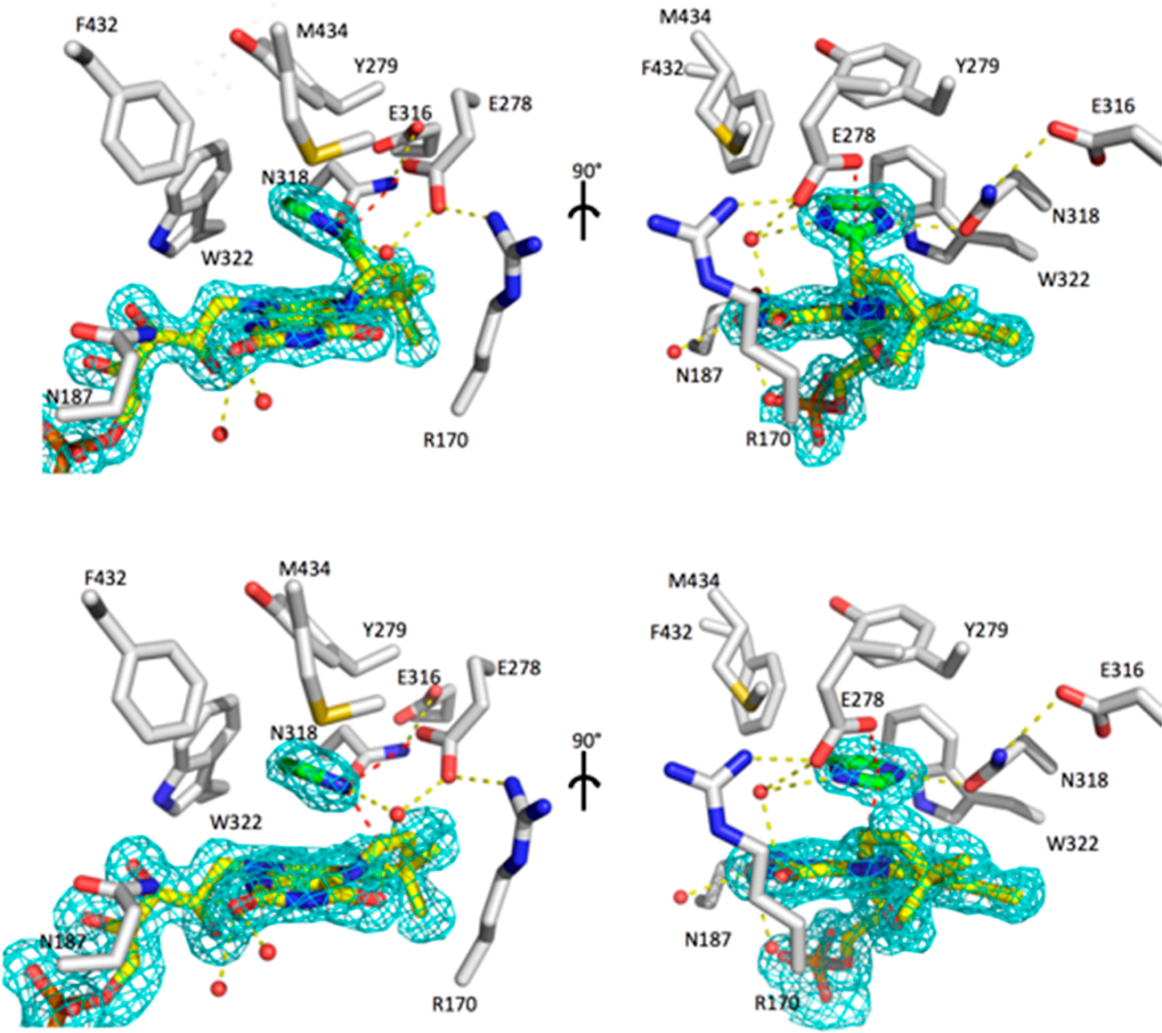
PA0254^UbiX^ active site structure. Active site residues shown in atom-colored sticks (gray carbons) with the prFMN^iminium^ cofactor shown with yellow carbons. Bound imidazole derived from the crystallization buffer is shown with green carbons. Omit *F*_o_*F*_c_ map corresponding to cofactor and imidazole is contoured at 3 sigma and shown as a cyan mesh. In two monomers a covalent bond is formed between imidazole C2 and the prFMN C1′ (top view), while the active site of the other monomers lacks electron density in between the imidazole and the prFMN, indicating a noncovalent complex. Hydrogen bonds are shown in yellow dotted lines, while the key Glu278 imidazole C2 and imidazole C2/prFMN C1′ interactions are shown in red.

The PA0254^UbiX^ active site is readily identified by the presence of the prFMN cofactor. In each of the four monomers, additional electron density was observed located above the prFMN cofactor (Figure 13). The density was modeled as an imidazole derived from the crystal mother liquor. In two of the PA0254^UbiX^ monomers, the imidazole density was continuous with the prFMN density, suggesting a covalent bond between the imidazole C2 and the C1′ of the prenyl-derived prFMN ring. The electron density in the other two monomers was noncontinuous between the imidazole and prFMN and thus modeled as a noncovalent, stacking interaction between imidazole and the prFMN aromatic plane. In both the covalent and the noncovalent ligand complexes, the imidazole N1 is within the hydrogen-bonding distance of the N318 amide side chain. The H-bonding interaction of N318 with E316 suggests that the N318 amide is indeed oriented with the oxygen toward the imidazole nitrogen. Hydrophobic interactions with Y279, W322, F432, and M434 largely occlude the imidazole from the solvent, with the exception of the water molecule hydrogen bonding to the imidazole N3. In the case of the covalent imidazole adduct, the imidazole C2 is positioned 3.2 Å away from the conserved E278. The latter has been implicated as the key active site acid–base catalyst required to either donate or abstract a proton from the prFMN-bound substrate. At 1.65 Å resolution it is not possible to directly determine the protonation state of the imidazole C2 for the covalent adduct. However, the fact that the C1′ is not in the plane with the imidazole moiety (a deviation of approximately 35°) suggests either protonation of C2 (i.e., sp^*3*^ hybridization, nonaromatic) or considerable strain on the aromatic sp^*2*^-hybridized C2 (i.e., Int 2-like) imposed by the protein active site. Previous studies on *A. niger* Fdc1 have confirmed that the active site is able to constrain prFMN adducts, thus controlling the internal thermodynamics of the reaction.^11^

### Reversible Binding of Imidazole to PA0254^UbiX^

**Figure 14:**
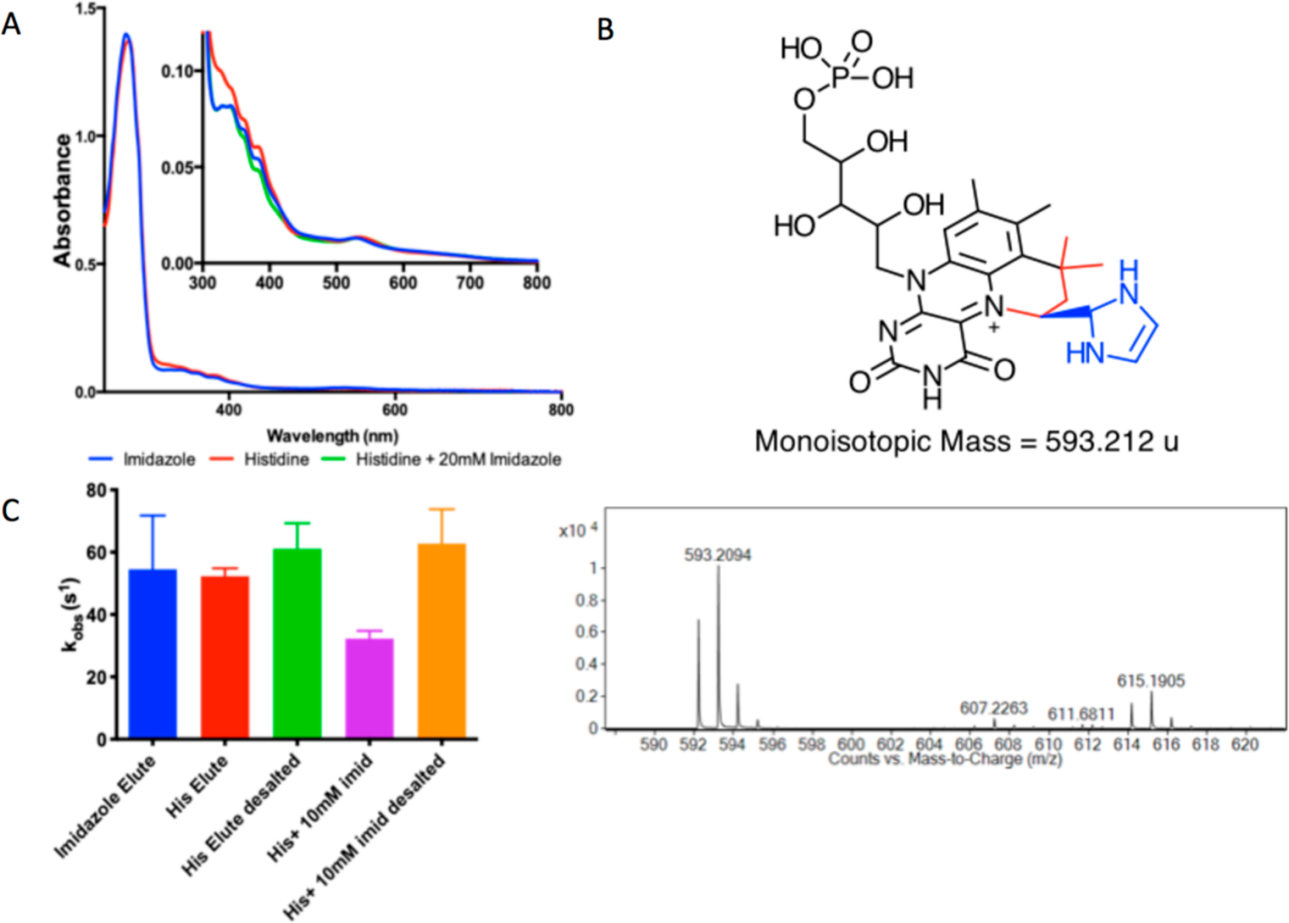
Imidazole binding to PA0254^UbiX^. (A) UV–vis spectra of PA0254^UbiX^ eluted from IMAC using imidazole (blue) or histidine (red). Spectrum of the histidine-eluted protein following addition of 20 mM imidazole and desalting is also shown (green). (B) Mass spectrum of low molecular weight species within PA0254^UbiX^ eluted with imidazole, indicating a species with a mass of 593.2094 Da, close in mass to the predicted covalent imidazole–prFMN adduct. (C) Activity of PA0254^UbiX^ against P2C following various treatments. Imidazole- and histidine-eluted proteins possess similar activities. Addition of 10 mM imidazole results in ∼50% inhibition; however, activity is recovered upon removal of excess imidazole (error bars represent SEM, *n* = 3).

Following the identification of the imidazole adduct in the PA0254^UbiX^ crystal structure, the enzyme was subsequently purified using histidine as elutant as opposed to imidazole. A comparison of the UV–vis profiles of the imidazole- and histidine-eluted enzymes revealed subtle differences with the imidazole-eluted protein having a slightly lower and more defined peak at ∼340 nm (Figure 14). Upon incubation of the histidine-eluted protein with imidazole, a small spectral shift was observed. Mass spectrometry of the imidazole-bound species revealed a peak at 593.2094 Da, close to the predicted mass of a covalently bound imidazole–prFMN adduct (593.2125 Da). Both imidazole and histidine purified proteins were found to have similar activities. The activity of the histidine-eluted protein was inhibited by ∼50% by addition of 10 mM imidazole. Full activity was recovered following removal of imidazole by desalting, suggesting that reversible prFMN adduct formation occurs.

### Mutagenesis of PA0254 Supports a Key Role for N318

**Figure 15:**
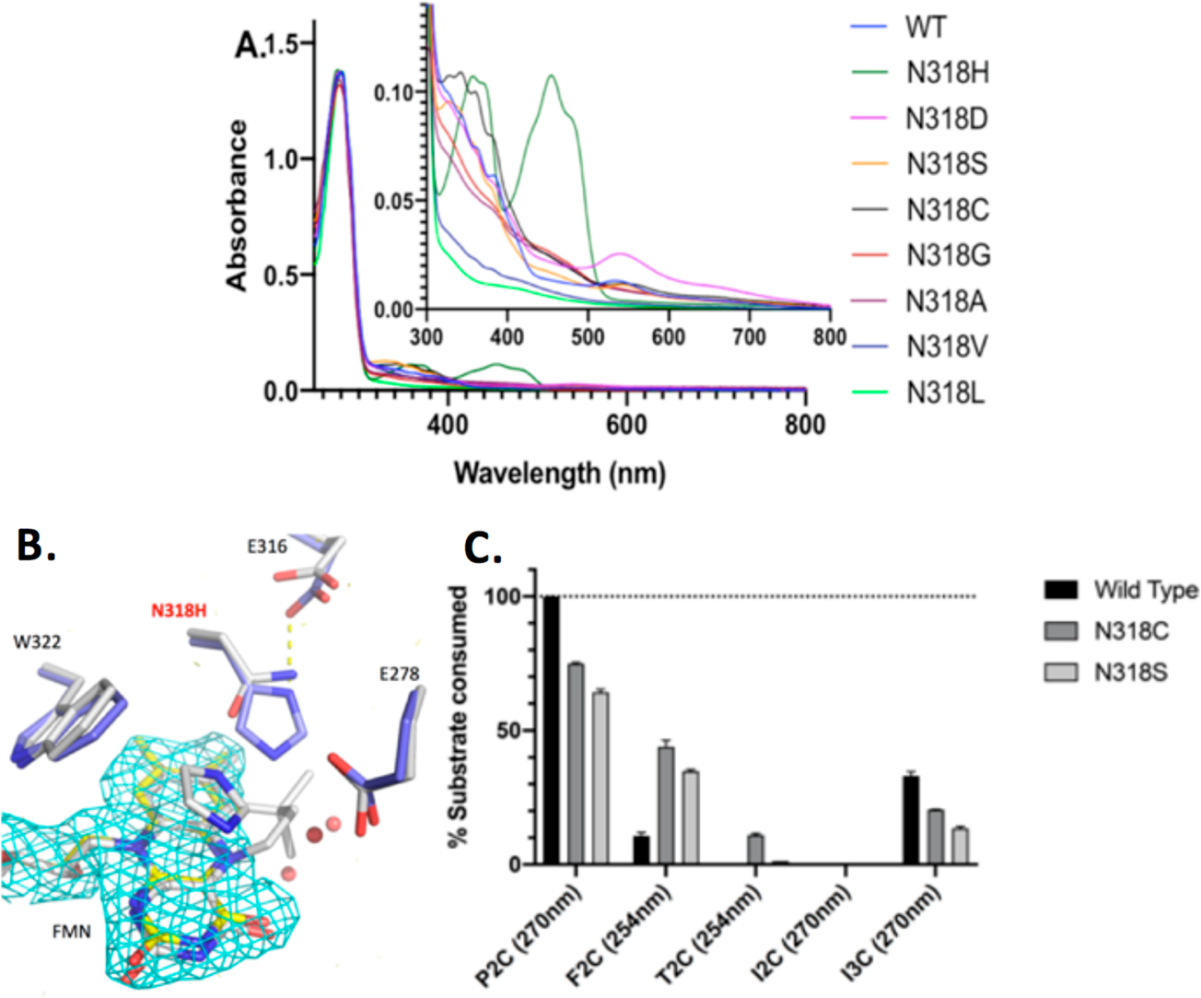
PA0254^UbiX^ N318X characterization. (A) UV–vis spectra of PA0254 variants normalized on the *A*_280_ peak. (Inset) Zoom into cofactor-related features in the 300–800 nm region. (B) Overlay of the WT PA0254^UbiX^ crystal structure (with gray carbons) with the N318H mutant (in blue carbons) reveals bound FMN (yellow carbons). Omit *F*_o_*F*_c_ map corresponding to FMN is contoured at 3 sigma and shown as a cyan mesh. (C) Comparison of substrate depletion levels following addition of and incubation with WT, N318C, and N318S variants for a range of heteroaromatic acids. Wavelength used for quantification shown in brackets. Error bars represent SEM, *n* = 3.

A number of PA0254 variants were generated in order to establish the proposed role of N318 in substrate recognition. In the case of HmfF, the corresponding residue is H297, which was established to be key to ensuring specificity for furan-type substrates. Hence, we designed a PA0235 N318H variant to test whether this would affect the substrate specificity and possibly alter the preference to furan-type substrates. Surprisingly, the purified N318H variant was found to possess an intense yellow color consistent with oxidized flavin (Figure 15). Structure determination of the N318H variant revealed that the introduced histidine side chain partially occupies the site of the prenyl-derived fourth prFMN ring, preventing prFMN binding. Hence, we created a series of distinct N318X variants, restricted to amino acids with a size similar to or smaller than asparagine. While all variants appeared to bind cofactor, this unfortunately occurs to varying levels as judged by the UV–vis spectra, making detailed comparison of the relative activity levels complicated. The PA0254^UbiX^ variants were screened for decarboxylation activity with P2C and furan-2-carboxylic acid (F2C). Only the WT and N318D enzymes were able to achieve complete decarboxylation of the P2C substrate. Of the variants tested, N318C and N318S also had considerable levels of activity whereas replacement of N318 with nonpolar residues resulted in very low conversion, possibly due to low cofactor incorporation. The N318S and N318C preparations yielded a higher conversion of the F2C and T2C substrate compared to the WT enzyme under the conditions tested.

### DFT Calculations Reveal Two Int1 Species

In order to better understand how the P2C, F2C, and T2C substrates react with the prFMN, density functional theory (DFT) calculations of prFMN adducts formed with P2C, F2C, T2C, Im2C, and Im4C were carried out. These calculations were performed in implicit water rather than the enzyme active site for simplicity and to facilitate the direct comparison of the role of the adduct moiety. In all cases, stable ring-open Int1^open^ C1′–C2 adducts associated with a nucleophilic or electrophilic addition process were observed. This species has been observed in previous DFT active site “cluster” calculations where decarboxylation appears to occur from this rather than the ring-closed Int1 species (Int1^closed^) shown in Figure 1.^11,15^ With the notable exception of the zwitteronic Im2C–H^+^ species (with protons on both N1 and N3), the Int1^closed^ species was found to be stable for all adducts and lower in energy compared to the corresponding Int1^open^ adduct (Table S1). A natural charge analysis shows that in all cases, except for Im2C–H^+^, there is partial electron transfer from the substrate to the prFMN to form Int1^open^. The Int1^closed^ adduct is subsequently formed by electron transfer back from the prFMN to the substrate moiety of the adduct (Table S2, Supporting Information). This is consistent with the formation of Int1^open^ occurring by nucleophilic attack from the substrate, except for nonsubstrate Im2C–H^+^ which acts as an electrophile. Subsequently, the substrate Int1^open^ adducts formed by nucleophilic attack appear to have Wheland intermediate character.

## Discussion

The unusual metamorphosis of flavin to prFMN^iminium^ alters the fundamental character of this cofactor. In contrast to the C4a/N5 focused reactivity of flavin, the N5–C6 prenylation and subsequent oxidative maturation of prFMN^iminium^ lead to a stabilized azomethine ylide species with a reactive C4a/N5/C1′ center.^8,41^ Assuming prFMN^iminium^ underpins catalysis in all UbiD enzymes, certain general principles are likely to apply across this ubiquitous microbial enzyme family. Arguably, the best-understood enzyme is the *A. niger* ferulic acid decarboxylase that acts predominantly on cinnamic acid-type substrates.^8,42,43^ In this case, sufficient evidence has accumulated that supports a reversible 1,3-dipolar cycloaddition mechanism underpinning the (de)carboxylation reaction.^11^ Chemical precedent exists for the reaction of cinnamic acid-type dipolarophiles with azomethine ylide species, and the proposed mechanism also provides an explanation for the need of the elaborate FMN to prFMN^iminium^ transformation.

However, the substrate scope of the wider UbiD family extends far beyond cinnamic acid substrates, including both heteroaromatic and aromatic acids.^6^ It is clear that the latter substrates have inherently different reactivity and impose distinct conformational and energetic challenges for the enzyme. In the case of *A. niger* ferulic acid decarboxylase, variants have been developed that accept (hetero)aromatic acids with low reactivity observed for 2-naphthoic acid.^15^ In this case, an electrophilic aromatic substitution process has been proposed with stabilization of the charge on the Wheland intermediate through stacking with the prFMN^iminium^. In contrast, the decarboxylation of 3,4-dihydroxybenzoic acid substrates by AroY is proposed to occur via a quinoid intermediate formed concomitant with prFMN C1′–substrate C alpha bond formation.^14^ Finally, reversible decarboxylation of furan dicarboxylic acid by HmfF has been proposed to occur through either a cycloaddition or an electrophilic aromatic substitution process.^13^

**Figure 16:**
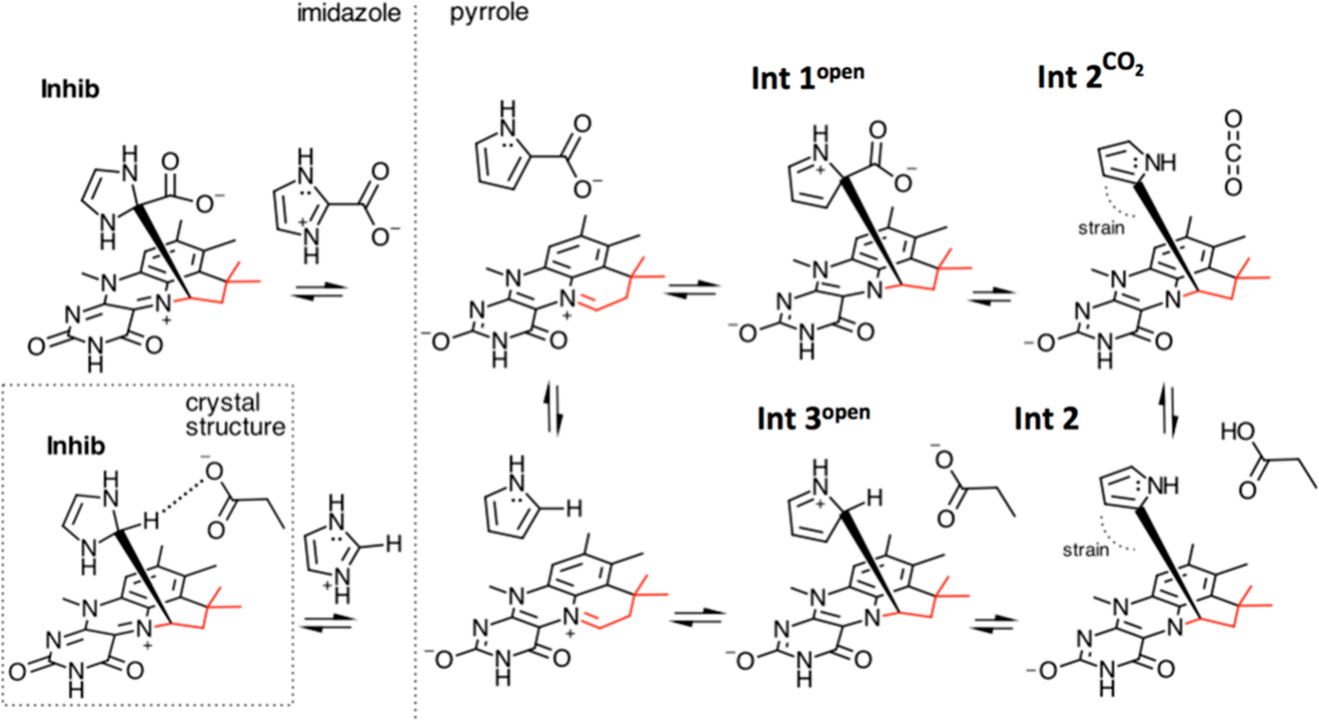
Proposed mechanism for PA0254/HudA. prFMN^iminium^ electrophilic aromatic substitution reaction with pyrrole underpins reversible decarboxylation, while nucleophilic addition to the imidazole C2 position leads to reversible inhibition.

In the case of PA0254, the structure of the covalent prFMN^iminium^ C1′–imidazole C2 adduct provides further insight into the general reaction of UbiD enzymes with heteroaromatic compounds. Crucially, the imidazole-2-carboxylic acid is not a substrate for the enzyme, while imidazole acts as a reversible inhibitor. The chemical reactivity of imidazole for electrophilic aromatic substitution is substantially lower than that of the corresponding pyrrole and only occurs on C4/C5 positions. In contrast, the imidazole C2 position is electron deficient and undergoes nucleophilic aromatic substitution when a suitable leaving group is present. This suggests that reversible bond formation with imidazole C2 might occur through nucleophilic attack of C1′ concomitant with protonation at N3. Crucially, this prFMN adduct species (labeled **Inhib** in Figure 16) would not support H/D exchange or (de)carboxylation at the C2 position, in line with our observations in solution.

In contrast, reaction with pyrrole/furan/thiophene compounds is likely to occur through electrophilic aromatic substitution at the C2 position via a Wheland-type intermediate Int1^open^/Int3^open^ (Figure 16). While DFT calculations indicate an Int1^closed^ species might occur, it is unclear what role it plays in catalysis. It is however interesting to note the Int1^closed^ species appears inaccessible to the zwitterionic imidazole-2-carboxylic acid. The Int1 and Int3 intermediates provide access to the central Int2 species via, respectively, (de)carboxylation and (de)protonation. It is possible that additional through-space electronic interactions with the prFMN^iminium^ stabilize the charge on the Wheland intermediate^44^ and assist with retaining the strained configuration of Int2. The latter configuration is required within the context of the closed active site as highlighted by the PA0254^UbiX^–imidazole complex. A configuration whereby the C1′ prFMN substituent is positioned in the plane of the substrate aromatic ring would require active site reorganization, similar to the domain motion observed when comparing the *apo*- and *holo*-forms of PA0254.

However, to ensure rapid turnover, highly stable covalent intermediates should be avoided, and we propose that the aromatic group in the Int2 adduct remains parallel rather than perpendicular to the prFMN^iminium^ plane. The trend in yields obtained with pyrrole-, furan-, and thiophene-2-carboxylic acid compounds mirrors the respective reactivity toward electrophilic aromatic substitution in solution. No activity with pyrrole-3-carboxylic acid could be detected, but the related indole-3-carboxylic acid readily yielded indole. This again mirrors the trends for electrophilic aromatic substitution reactivity, which is preferred for pyrrole at the 2 position while indole occurs at the 3 position. Unfortunately, no reaction could be observed with benzoic acid, a substrate that arguably presents the most formidable barrier due to the high aromaticity of the benzene ring. However, UbiD enzymes have been implicated in microbial anaerobic benzene degradation where carboxylation is proposed to activate the substrate for further degradation.^45^ We have not been able to establish whether benzoic acid can bind to PA0254^UbiX^, while variants aimed at creating a more hydrophobic active site (i.e., N318A/V/L) did not readily bind prFMN. It thus remains possible that a UbiD enzyme with an active site optimized for benzene/benzoic acid binding might be able to catalyze electrophilic aromatic substitution at rates sufficient to support the relatively slow microbial growth seen during anaerobic benzene degradation. Furthermore, the domain dynamics indirectly observed here, but not invoked for either Fdc1 or PA0254 reaction, might couple to the reaction coordinate in the case of more challenging transformations such as benzene/napthalene or phenylphosphate carboxylation.^29,45,46^

While the exact biological role of PA0254/HudA as a pyrrole-2-decarboxylase is yet to be established, the recent observation that P2C eliminates the expression of quorum sensing cascade and pathogenic factors of *P. aeruginosa* PAO1 on both phenotypic and genotypic levels^47^ suggests that it could be involved in P2C detoxification. Previous identification of PA0254/HudA as a virulence attenuation factor, on the other hand,^21^ might indicate that the product of the decarboxylation reaction, pyrrole, is responsible for the observed effects.

## Supporting Information Available

HF energy (hartrees) computed for the Int1^closed^ adducts; natural charges computed for the substrate moiety; alignment of the Int1^open^ and Int1^closed^; DFT models used in this study (PDF)
