# Supplementary material for: Structure and Mechanism of Pseudomonas aeruginosa PA0254/HudA, a prFMN-Dependent Pyrrole-2-carboxylic Acid Decarboxylase Linked to Virulence
Source: ACS Catal. 2021 Feb 17;11(5):2865–78. doi: 10.1021/acscatal.0c05042 (PMC7976604; doi:10.1021/acscatal.0c05042)
Supplement: Supplementary file 1 — cs0c05042_si_001.pdf [file cs0c05042_si_001.pdf]

Supporting information

Structure and mechanism of *Pseudomonas aeruginosa* PA0254/HudA, a prFMN-dependent pyrrole-2-carboxylic acid decarboxylase linked to virulence

Karl A.P. Payne[1$], Stephen A. Marshall[1], Karl Fisher[1], Stephen E.J. Rigby[1], Matthew J. Cliff[1], Reynard Spiess[1], Diego M. Cannas[2], Igor Larrosa[2], Sam Hay[1], David Leys[1*]

[1]Manchester Institute of Biotechnology, University of Manchester, 131 Princess Street, Manchester, M1 7DN, UK

[2]Department of Chemistry, University of Manchester, Chemistry Building, Oxford Road, Manchester, M13 9PL, UK

[$]present address :FBRH, University of Manchester, 131 Princess Street, Manchester, M1 7DN, UK

*david.leys@manchester.ac.uk

DFT Supporting Information:

Table S1. HF energy (hartrees) computed for the $Int1^{closed}$ adducts of P2C, F2C, T2C, Im2C and Im4C with prFMN in implicit water.

	$Int1^{open}$	$Int1^{closed}$		$Int1^{open}$–$Int1^{closed}$ (kJ/mol)
P2C	-1580.872256	-1580.875149		+7.6
F2C	-1600.725775	-1600.738391		+33.1
T2C	-1923.710723	-1923.721422		+28.1
Im2C	-1596.912481	-1596.897529		-39.3*
$Im2C\text{-}H^+$	-1597.369815	Not stable		-
Im4C	-1596.921557	-1596.93624		+38.5

* note that only Im2C has a more stable $Int1^{open}$ adduct relative to $Int1^{closed}$.

Table S2. Natural charges computed for the substrate moiety of the $Int1^{open}$ and $Int1^{closed}$ adducts of P2C, F2C, T2C, Im2C and Im4C with prFMN in implicit water.

	$Int1^{open}$	$Int1^{closed}$	$Int1^{open} - Int1^{closed}$
P2C	-0.36	-0.90	+0.53
F2C	-0.56	-0.91	+0.36
T2C	-0.62	-0.93	+0.31
Im2C	-0.49	-1.13	+0.65
Im2C-H^+	-0.10*	Not stable	-
Im4C	-0.50	-0.91	+0.41

* note that Im2C-H^+ has a neutral charge, whereas all other substrates/inhibitors have a charge of -1.

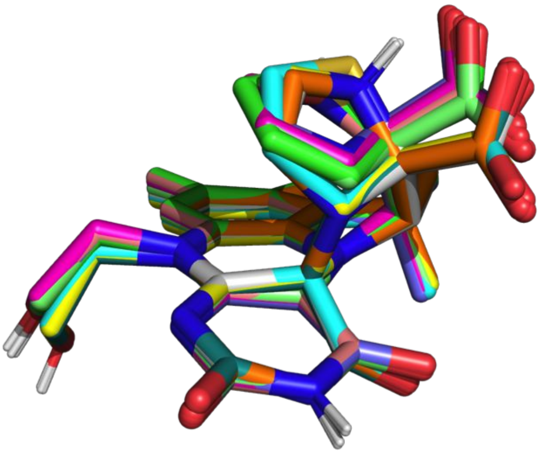

Figure S1. Alignment of the $Int1^{open}$ and $Int1^{closed}$ prFMN- P2C, F2C, T2C, Im2C and Im4C species.

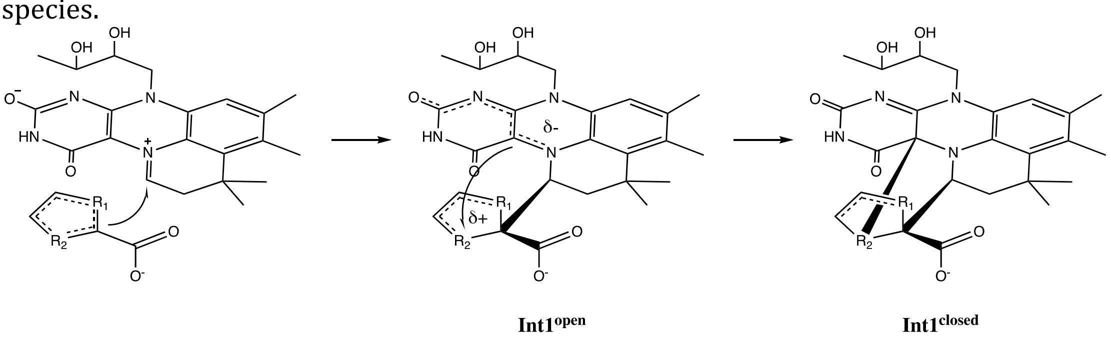

Scheme S1. The DFT models used in this study, showing a 2-step mechanism for the formation of Int 1. In all cases except for protonated Im2C, there is partial electron transfer from the substrate to the prFMN to form the ring-open adduct. The ring closed adduct is formed by electron transfer back from the prFMN to the substrate moiety of the adduct.
